# Serum tumour marker CA 125 in monitoring of ovarian cancer during first-line chemotherapy

**DOI:** 10.1054/bjoc.2001.1787

**Published:** 2001-05

**Authors:** M K Tuxen, G Sölétormos, P Dombernowsky

**Affiliations:** Department of Oncology, Herlev Hospital, University of Copenhagen, 2730 Herlev; Department of Internal Medicine, Hvidovre Hospital, 2650 Hvidovre; Department of Clinical Biochemistry, Hillerd County Hospital, 3400 Hillerd, Denmark

**Keywords:** CA 125, ovarian cancer, progression criteria, monitoring, biological variation, analytical variation

## Abstract

The value of the serum tumour marker CA 125 to date has been in the monitoring of ovarian cancer patients for response to therapy and for recurrence of disease. However, despite the availability of serial data on CA 125, the problem of interpreting a change over time is still unsolved. The aim of this study was to assess the ability of CA 125 to monitor patients with ovarian cancer during postoperative chemotherapy. 255 patients with stage IC-IV ovarian cancer were allocated to the tumour marker monitoring study. The evaluation of CA 125 information was based on the analytical imprecision, the normal intra-individual biological variation, the sampling interval, and the cut-off value. Additionally, a new assessment criterion based upon an increment of 2.5 times the baseline CA 125 concentration confirmed by a third measurement was elaborated and the utility investigated. The efficiency of CA 125 for identifying progression and non-progression during first-line chemotherapy was 91.9%. The median lead time for true positive results was 41 days. Using the new elaborated criterion the efficiency of CA 125 for identifying progression and non-progression during first-line chemotherapy was 90.5%. The median lead time for true positive results was 35 days. CA 125 gave reliable prediction of progressive disease during postoperative chemotherapy. The results indicate a high applicability of the presented progression criteria during CA 125 monitoring of patients with changing activity of ovarian cancer. © 2001 Cancer Research Campaign www.bjcancer.com


*British Journal of Cancer* (2001) **84**(10), 1301–1307
© 2001 Cancer Research Campaign
doi: 10.1054/ bjoc.2001.1787, available online at http://www.idealibrary.com on IDEAL®

http://www.bjcancer.com

### Serum tumour marker CA 125 in monitoring of ovarian cancer during first-line chemotherapy

**MK Tuxen[1,2], G Sölétormos[3] and P Dombernowsky[1]**

Department of Oncology[1], Herlev Hospital, University of Copenhagen, 2730 Herlev; Department of Internal Medicine[2], Hvidovre Hospital, 2650 Hvidovre; Department of Clinical Biochemistry[3], Hillerød County Hospital, 3400 Hillerød, Denmark

**Summary** The value of the serum tumour marker CA 125 to date has been in the monitoring of ovarian cancer patients for response to therapy and for recurrence of disease. However, despite the availability of serial data on CA 125, the problem of interpreting a change over time is still unsolved. The aim of this study was to assess the ability of CA 125 to monitor patients with ovarian cancer during postoperative chemotherapy. 255 patients with stage IC-IV ovarian cancer were allocated to the tumour marker monitoring study. The evaluation of CA 125 information was based on the analytical imprecision, the normal intra-individual biological variation, the sampling interval, and the cut-off value. Additionally, a new assessment criterion based upon an increment of 2.5 times the baseline CA 125 concentration confirmed by a third measurement was elaborated and the utility investigated. The efficiency of CA 125 for identifying progression and non-progression during first-line chemotherapy was 91.9%. The median lead time for true positive results was 41 days. Using the new elaborated criterion the efficiency of CA 125 for identifying progression and non-progression during first-line chemotherapy was 90.5%. The median lead time for true positive results was 35 days. CA 125 gave reliable prediction of progressive disease during postoperative chemotherapy. The results indicate a high applicability of the presented progression criteria during CA 125 monitoring of patients with changing activity of ovarian cancer. © 2001 Cancer Research Campaign http://www.bjcancer.com

**Keywords**: CA 125; ovarian cancer; progression criteria; monitoring; biological variation; analytical variation

Ovarian cancer is the fourth most frequent cause of cancer death in women and the most fatal gynaecologic malignancy (Storm et al, 1993). At the time of diagnosis approximately 70% to 75% of the patients already have advanced disease (Thigpen et al, 1995), evidenced by involvement of pelvic and abdominal organs or of more distant sites. Current standard treatment of advanced ovarian carcinoma consists of aggressive primary cytoreductive surgery followed by systemic platinum-based combination chemotherapy. After cytoreductive surgery, most patients maintain small amounts of residual tumour at multiple sites throughout the peritoneal cavity, which is difficult to evaluate by physical examination and imaging techniques during subsequent treatment.

To monitor ovarian cancer patients serum tumour marker CA 125 has been proposed as a supplement to other non-invasive diagnostic methods. An important characteristic of CA 125 is the ability to reflect changes in tumour mass during chemotherapy or in the follow-up period after completion of therapy. If patients have elevated serum CA 125 levels at diagnosis, serial serum CA 125 determinations during initial therapy accurately reflect the disease course in more than 74% of the matched events (clinical versus marker response, stability or progression) (Tuxen et al, 1995). However, there is no consensus concerning the interpretation of consecutive tumour marker concentrations and several different criteria have been proposed (Bast et al, 1983, 1984; Krebs et al, 1986; Fioretti et al, 1987; Panza et al, 1988; Gadducci et al, 1990, 1991; Cruickshank et al, 1992; Fioretti et al, 1992; Rustin et al, 1992; Vergote et al, 1992; Rustin et al, 1993, 1997), but none are able to eliminate the false positive marker signals as regards tumour progression.

The present study was performed to improve the clinical value of CA 125 monitoring by introduction of a precise definition of CA 125 progression. We investigated the ability of the serum tumour marker CA 125 to monitor patients with ovarian cancer during first-line chemotherapy when the evaluation of CA 125 information was based on the analytical imprecision and the normal intra-individual biological variation. Additionally, we compared this approach with other methods of interpreting changes in CA 125 concentrations. The study was based on our previously described model for the interpretation of serum CA 125 results based on the analytical imprecision and the average inherent intra-individual biological variation (Tuxen et al, 2000).

*Received 26 September 2000*
*Revised 24 October 2000*
*Accepted 9 February 2001*

*Correspondence to:* MK Tuxen

### MATERIALS AND METHODS

### Patients

255 patients with stage IC-IV ovarian cancer were allocated to the tumour marker monitoring study. All participated in the North Thames Ovary Trial, England, of 5 versus 8 courses of carboplatin 400 mg/m$^2$ or cisplatin 75 mg/m$^2$ every 4 weeks. The inclusion ranged from December 1989 to April 1994. The North Thames Ovary Trial was approved by the regional Ethical Committees. 48 patients were ineligible for the present study due to early death (death within the first 4 weeks of treatment) in 3 patients, another primary cancer in 11, and treatment with monoclonal antibody, which could cause falsely elevated CA 125 levels (Boscato and

Stuart, 1988; Nicholson et al, 1996) in 13. 18 patients had insufficient sampling (<3 CA 125 samples) and in 3 the last sample was taken more than 3 months prior to clinical progressive disease (clinical PD). Thus, 207 patients were considered eligible for tumour marker assessment.

### Analytical methods

A total of 1366 CA 125 samples were obtained during first-line chemotherapy with a median of 6 samples per patient. Each sample from one individual patient was assayed in duplicate in different assay runs.

Serum CA 125 was initially determined by ELSA-CA 125, an immunoradiometric assay from CIS Bio International (Gif-sur-Yvette Cédex, France). The applied cut-off value was 35 U/mL as recommended by Bast et al (1983) and the manufacturer (personal communication).

Since August 1992, CA 125 has been measured by the Cobas Core CA 125 II EIA assay, a one-step solid-phase enzyme immunoassay based on the sandwich principle from Roche Diagnostic Systems (Basel, Switzerland). The cut-off value recommended by the manufacturer was 35 U/mL (Roche Diagnostic Systems, 1996).

### Procedure for matching clinical and CA 125 information and definitions of CA 125 progression criteria

The results of the tumour marker assessment were compared retrospectively with the clinical data. No clinical evaluation of the status of the disease was performed during first-line chemotherapy. However, if a patient developed signs of clinical PD, appropriate clinical examinations were performed and the date of progression registered. Thus, only data of clinical PD were available for first-line chemotherapy.

A change between 2 consecutive marker concentrations during monitoring of patients with ovarian cancer is statistically significant, if the difference (the critical difference) exceeds $\sqrt{2} \times Z \times \sqrt{(CV_A^2 + CV_I^2)}$ (Fraser et al, 1990; Fraser and Petersen, 1991). $\sqrt{2}$ is a constant (2 measurements). Z is the Z-statistic, which depends on the probability selected for significance and on whether the change expected is unidirectional (only one option, either an increment or a decrement) or bidirectional (2 options because it is unknown whether the concentration will rise or fall). Z equals 1.65 for unidirectional and 1.96 for bidirectional changes, for which less than 5% of differences will exceed this value during steady state conditions. $CV_I$, which is the average intra-individual biological variation of CA 125 was calculated previously on basis of 25 patients with both clinical disease and marker concentrations in a steady state. The $CV_I$ was 24% (Tuxen et al, 2000). $CV_A$, the analytical imprecision corresponding to a patient's CA 125 baseline concentration, was read from the respective precision profiles of the total analytical variation for CA 125. The total analytical variation comprised both the intra- and the inter-assay variation. The baseline concentration is a CA 125 concentration, from which an increment or a decrement starts. According to this approach the critical difference is variable and depends on the analytical imprecision at the considered concentration level.

Assessment of CA 125 data was based on the magnitude of the critical difference, the duration of the difference, and the cut-off value (Sölétormos et al, 1993a, 1996). The duration of the difference was decided to be of at least 28 days due to a practical approach as the chemotherapy cycles were given every 4 weeks and CA 125 samples normally taken on the day of treatment. Thus, the criteria for marker PD depended on whether the marker increment started below or above the cut-off value.

*Criterion 1*: for increment starting below the cut-off value the criteria for CA 125 PD were: a significant increment from below to above the cut-off value during the first time interval of ≥ 28 days. The increment during the first time interval was confirmed by a further measurement during the second time interval of ≥ 28 days. It was not required that a further increase during the second time interval significantly exceeded the concentration obtained during the first time interval (Figure 1A).

*Criterion 2*: for increment starting above the cut-off value the criteria for CA 125 PD were: an increment from the baseline concentration during the first time interval of ≥ 28 days, which did not have to be significant. However, the concentration obtained during the second time interval of ≥ 28 days should significantly exceed the baseline concentration (Figure 1B).

The calculated date of marker PD was the date of the first sample, which indicated progression: the second sample if the critical difference was achieved during the first time interval (Figure 1A and 1B), and the third sample if the critical difference was achieved during the second time interval (Figure 1B). For the sake of simplicity these two criteria will hereafter be called 'the progression criteria 1 and 2'. CA 125 data were defined as non-assessable when all concentrations remained below the cut-off value or fluctuated across the cut-off value without achieving the critical difference (Figure 1C).

Beside the above-mentioned criteria, the performance of an alternative criterion based upon an increment of 2.5 times the baseline concentration was also assessed (Figure 2). According to this criterion CA 125 predicted progression if the baseline concentration increased 2.5 times during the first time interval of ≥28 days and the increase continued during the second time interval of ≥28 days. If the baseline concentration was below the cut-off value, the increment during the first time interval had to exceed the cut-off value. There was no requirement as regards the magnitude of the increment obtained during the second time interval. The increase of 2.5 times the baseline concentration was equal to the critical difference of approximately 86% as a change between two CA 125 concentrations, $x_1$ and $x_2$, was calculated as $(x_2 - x_1)/\sqrt{(x_1+x_2)}$. The calculated date of marker PD was the date of the second sample. For the sake of simplicity this criterion will hereafter be called 'the elaborated progression criterion'. As previously, CA 125 data were defined as non-assessable when all concentrations remained below the cut-off value or fluctuated across the cut-off value without fulfilling the progression criterion (Figure 1C).

### Statistics

Confidence intervals for frequencies were calculated according to Armitage and Berry (1994).

### RESULTS

### The progression criteria 1 and 2

The clinical and marker data for all patients were updated in April 1998. The pretreatment characteristics of the 207 eligible patients

 © 2001 Cancer Research Campaign are listed in Table 1. According to the CA 125 progression criteria 1 and 2, 16 patients were found marker non-assessable (Figure 1C). Additionally, the CA 125 profiles of 18 patients with clinical PD during the study period did not fulfil the progression criteria (Figure 1A and 1B) as the patients had an increment of CA 125 concentrations exceeding the critical difference but without a confirmatory sample due to discontinuation of marker monitoring or due to start of second-line treatment. These 16 patients were also excluded from the marker analysis. Of the remaining 173 assessable patients, 24 developed clinical PD during first-line chemotherapy. The distribution of all patients evaluated for entry into the CA 125 monitoring study is presented in Table 2.

The results of the matching procedure of clinical and CA 125 information obtained during first-line chemotherapy are shown in Table 3. CA 125 correctly identified 45.8% of the patients with, and 99.3% of the patients without clinical PD. A patient with marker PD had a 91.7% probability of developing clinical PD, whereas a patient without marker PD had a 91.9% probability of being without clinical PD. CA 125 gave a false-positive prediction of progression in one patient during treatment. The patient had an unusual marker profile with considerably fluctuating CA 125 levels during the whole study period. CA 125 concentrations ranged from 56 to 185 U/mL$^{-1}$ during chemotherapy and from 45 to 130 U/mL during follow-up. Despite relatively high marker concentrations, the patient did not experience clinical PD during first-line chemotherapy or during a subsequent follow-up period of 40.4 months.

Marker PD preceded clinical PD in 10 patients with a median positive lead time of 48.5 days (range 1–79) (Table 2). One patient had no lead time as the date of marker PD coincided with the date of clinical PD. In additional 2 patients, clinical PD preceded marker PD with negative lead times of 11 and 132 days, respectively. However, CA 125 was not measured at the time of clinical PD in the patient with a negative lead time of 11 days.

### Discarded criteria

In a number of previous publications, changes in serum CA 125 results were considered significant if the prechange concentration increased by 50% (Krebs et al, 1986; Vergote et al, 1992) or by

**Table 1** Pretreatment characteristics of 207 ovarian cancer patients eligible for CA 125 monitoring during first-line chemotherapy

| Characteristic | Number of patients (% of total) |
|---|---|
| Age | median 62 years (range 17–79 years) |
| Performance status | |
| 0 | 129 (62.3) |
| 1 | 71 (34.3) |
| 2 | 7 (3.4) |
| FIGO stage | |
| IC | 22 (10.6) |
| IIA | 1 (0.5) |
| IIB | 13 (6.3) |
| IIC | 14 (6.8) |
| IIIA | 3 (1.4) |
| IIIB | 14 (6.8) |
| IIIC | 117 (56.5) |
| IV | 23 (11.1) |
| Histologic type | |
| Serous | 92 (44.4) |
| Mucinous | 14 (6.8) |
| Endometrioid | 26 (12.6) |
| Clear cell | 17 (8.2) |
| Undifferentiated | 26 (12.6) |
| Mixed | 15 (7.2) |
| Unclassified | 17 (8.2) |
| Grade | |
| Well differentiated (grade I) | 22 (10.6) |
| Moderately differentiated (grade II) | 58 (28.0) |
| Poorly differentiated (grade III) | 71 (34.3) |
| Unclassified | 7 (3.4) |
| Well to moderately differentiated (grade I–II) | 10 (4.8) |
| Moderately to poorly differentiated (grade II–III) | 29 (14.0) |
| Well to poorly differentiated (grade I–III) | 3 (1.4) |
| Borderline | 7 (3.4) |
| Primary residual tumour size | |
| No residual tumour, negative cytology | 14 (6.8) |
| No residual tumour, positive cytology | 15 (7.2) |
| No residual tumour, cytology unknown | 12 (5.8) |
| <2 cm residual disease, single site | 10 (4.8) |
| <2 cm residual disease, multiple sites | 70 (33.8) |
| ≥2 cm residual disease | 60 (29.0) |
| Inoperable | 26 (12.6) |

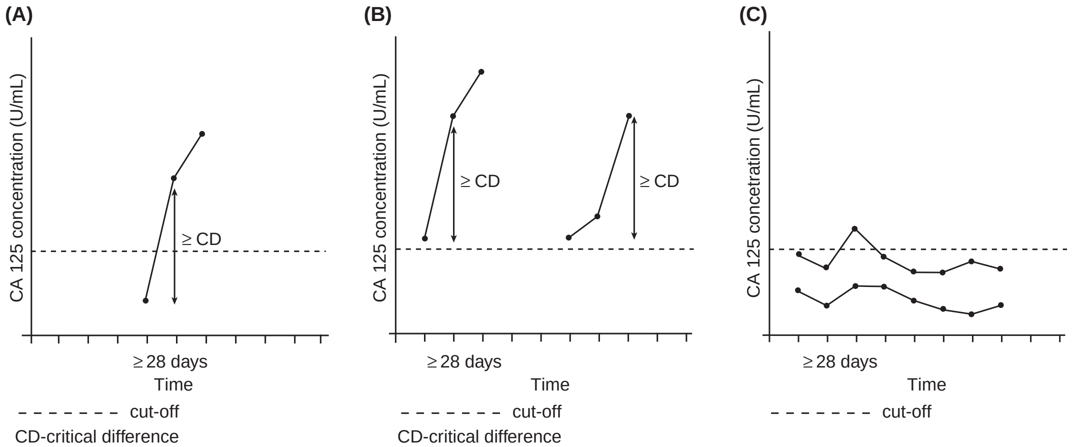

**Figure 1** Progression criteria for the serum tumour marker CA 125. **(A)** For increment starting below the cut-off value a significant increment from the baseline concentration occurred during the first time interval of ≥ 28 days and was confirmed by a further measurement during the second time interval of ≥ 28 days. The significant increment during the first time interval had to exceed the cut-off value. The significant increment was not required for the second time interval. **(B)** For increment starting above the cut-off value an increment obtained during the first time interval of ≥ 28 days did not have to be significant. However, a further measurement during the second time interval of ≥ 28 days had to significantly exceed the baseline concentration. **(C)** Non-assessable CA 125 data: all concentrations remained below the cut-off value or fluctuated across the cut-off value

© 2001 Cancer Research Campaign

**Table 2** The distribution of 255 patients with ovarian cancer allocated to the CA 125 monitoring study. Marker assessment was based on the progression criteria 1 and 2

| | | |
|---|---|---|
| 255 patients entered the North Thames Ovary Trial | → | 48 patients were ineligible for CA 125 assessment:<br>3 patients – early death<br>11 patients – another primary cancer<br>13 patients – treatment with monoclonal antibody<br>18 patients – insufficient sampling (<3 samples)<br>3 patients – last sample drawn more than 3 months prior to clinical PD |
| ↓ | | |
| 207 patients were eligible for CA 125 assessment | → | 16 patients were non-assessable because CA 125 concentration continuously remained below the cut-off value (2 patients with and 14 patients without clinical PD during first-line therapy).<br>18 patients with clinical PD were non-assessable because no confirmatory sample was measured (2 patients with and 16 patients without clinical PD during first-line therapy) |
| ↓ | | |
| 173 assessable patients were monitored during first-line chemotherapy | → | 24 patients developed clinical PD:<br>10 patients – marker PD with a positive lead time<br>1 patient – marker PD with zero lead time<br>1 patient – marker PD with a negative lead time as no measurement was taken at the time of clinical PD<br>1 patient – marker PD with a negative lead time due to clinical PD preceding marker PD<br>11 patients – never marker PD |

clinical PD – clinical progressive disease; marker PD – marker progression.

**Table 3** The ability of serum CA 125 to predict progressive disease during first-line chemotherapy among 173 ovarian cancer patients if marker assessment was based upon the progression criteria 1 and 2

| Characteristic | CA 125 |
|---|---|
| TP | 11 |
| FN | 13 |
| TN | 148 |
| FP | 1 |
| SE | 45.8 [25.9–65.8] |
| SP | 99.3 [96.3–99.9] |
| PPV | 91.7 [61.5–99.8] |
| NPV | 91.9 [87.7–96.1] |
| E | 91.9 [87.8–96.0] |
| Median lead time for true positive results, days (range) | 41 (0 to + 79) |

TP – true positive results; FP – false positive results; TN – true negative results; FN – false negative results; SE – sensitivity (%); SP – specificity (%); PPV – positive predictive value (%); NPV – negative predictive value (%); E – efficiency (%); [] – two-sided 95% confidence intervals (%). 24 patients developed clinical progressive disease during first-line chemotherapy.

100% (Bast et al, 1983, 1984; Fioretti et al, 1987; Panza et al, 1988; Gadducci et al, 1990, 1991; Cruickshank et al, 1992; Fioretti et al, 1992). However, these criteria produce too many false-positive results because in many cases the increase does not exceed the fluctuations attributable to analytical and biological variability, e.g. the increase is lower than the critical difference calculated on basis of biological and analytical variation in these cases.

As the doubling of the baseline CA 125 concentration is not always significant, an increment of 2.5 times the prechange concentration has also been considered. An increment of 2.5 times the prechange concentration invariably exceeds the critical difference. The criterion may, however, produce a false-positive prediction of progression in many patients as single increment of CA 125 may be misleading.

### An increment of 2.5 times the baseline concentration confirmed by a third measurement

The CA 125 progression criterion based upon an increment of 2.5 times the baseline concentration was further developed using 2 increasing CA 125 values, the second one for a confirmation of progression (Figure 2). This approach was developed in an attempt to follow the recommendations of the consensus committee on advanced epithelial ovarian cancer who dissuaded clinical decision making based on interpretation of a single CA 125 increment (Berek et al, 1999), because a single elevated concentration may be caused by an unspecific fluctuation not related to a change in disease activity.

Out of 207 patients eligible for CA 125 assessment 39 patients were excluded from the marker analysis, 16 due to a non-assessable marker profile (Figure 1C) and 23 due to a missing confirmatory sample. The patients from the latter group, all with clinical PD during the study period, had an increment of CA 125 concentrations exceeding an increment of 2.5 times the baseline concentration but were without a confirmatory sample measured due to discontinuation of marker monitoring or due to start of second-line treatment. Among the remaining 168 assessable patients, 24 developed clinical PD during first-line chemotherapy. The distribution of all patients evaluated for entry into the CA 125 monitoring study is presented in Table 4.

The ability of serum CA 125 to predict progressive disease during first-line chemotherapy is presented in Table 5. There was a 33.3% and 100.0% concordance between the clinical and marker information in patients with and without clinical PD, respectively. No false-positive and 16 false-negative results were obtained during treatment. Therefore, marker PD was associated with a 100.0% probability of clinical PD, whereas CA 125 concentrations remaining below the cut-off value were associated with a 90.0% probability of being without clinical PD.

Among the 24 patients with clinical PD, 7 patients had early marker PD with a median positive lead time of 41 days (range

*© 2001 Cancer Research Campaign*

**Table 4** The distribution of 255 patients with ovarian cancer allocated to the CA 125 monitoring study. Marker assessment was based upon an increment of 2.5 times the baseline concentration confirmed by a third measurement

| | | |
|---|---|---|
| 255 patients entered the North Thames Ovary Trial | → | 48 patients were ineligible for CA 125 assessment:<br>3 patients – early death<br>11 patients – another primary cancer<br>13 patients – treatment with monoclonal antibody<br>18 patients – insufficient sampling (< 3 samples)<br>3 patients – last sample drawn more than 3 months prior to clinical PD |
| ↓ | | |
| 207 patients were eligible for CA 125 assessment | → | 16 patients were non-assessable because CA 125 concentrations continuously remained below the cut-off value (2 patients with and 14 patients without clinical PD during first-line therapy).<br>23 patients with clinical PD were non-assessable because no confirmatory sample was measured (2 patients with and 21 patients without clinical PD during first-line therapy). |
| ↓ | | |
| 168 assessable patients were monitored during first-line chemotherapy | → | 24 patients developed clinical PD:<br>7 patients – marker PD with a positive lead time<br>1 patient – marker PD with zero lead time<br>2 patients – marker PD with a negative lead time due to clinical PD preceding marker PD<br>14 patients – never marker PD |

clinical PD – clinical progressive disease; marker PD – marker progression.

**Table 5** The ability of serum CA 125 to predict progressive disease during first-line chemotherapy among 168 ovarian cancer patients if marker assessment was based upon an increment of 2.5 times the baseline concentration confirmed by a third measurement

| Characteristic | CA 125 |
|---|---|
| TP | 8 |
| FN | 16 |
| TN | 144 |
| FP | 0 |
| SE | 33.3 [15.6–55.3] |
| SP | 100.0 [> 97.9]* |
| PPV | 100.0 [> 68.8]* |
| NPV | 90.0 [85.4–94.6] |
| E | 90.5 [86.0–94.9] |
| Median lead time for true positive results, days (range) | 35 (0 to + 70) |

TP – true positive results; FP – false positive results; TN – true negative results; FN – false negative results; SE – sensitivity (%); SP – specificity (%); PPV – positive predictive value (%); NPV – negative predictive value (%); E – efficiency (%); [] – two-sided 95% confidence intervals (%); * – one-sided 95% confidence intervals (%). 24 patients developed clinical progressive disease during first-line chemotherapy.

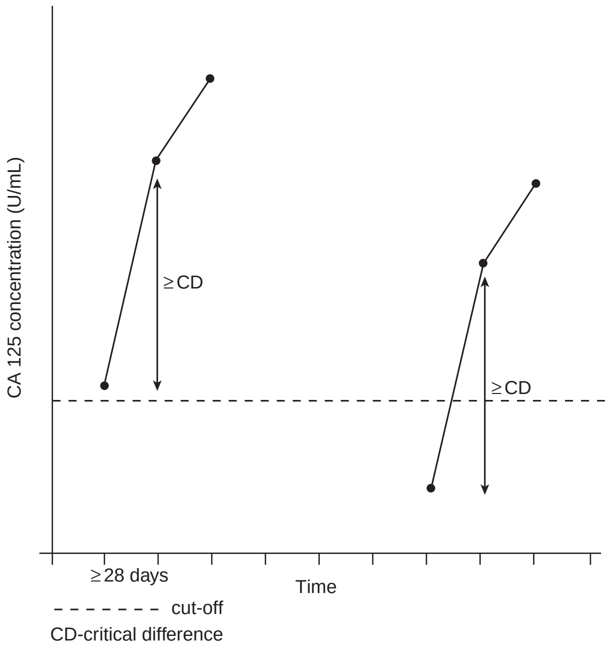

**Figure 2** The progression criterion for the serum tumor marker CA 125 based upon an increment of 2.5 times the baseline concentration confirmed by a third measurement. An increment of 2.5 times the baseline concentration occurred during the first time interval of ≥ 28 days and an increase continued during the second time interval of ≥ 28 days. If the baseline concentration was below the cut-off value, the increment during the first time interval had to exceed the cut-off value. There was no requirement as regards the magnitude of the increment obtained during the second time interval

1–70); in one patient with no lead time CA 125 became elevated at the time of clinical PD (Table 4). In 2 patients CA 125 increased after the detection of progression by other techniques giving negative lead times of 21 and 167 days, respectively.

### DISCUSSION

The consensus committee on advanced epithelial ovarian cancer has recently stated that the tumour marker CA 125 has the potential to play an important role in individual patient management (Berek et al, 1999). The marker may be: (1) an accurate early indicator of treatment failure during front-line therapy, (2) of value in confirmation of relapse, and (3) used during relapse therapy as an aid to decision making about continuation of therapy. However, there is no consensus concerning the interpretation of serial CA 125 measurements. Therefore, if CA 125 is to be implemented in routine clinical situations, a precise definition of CA 125 response and progression is necessary.

In previous publications changes in CA 125 concentrations have usually been evaluated with reference to empirical criteria, such as an arbitrary percentage of change between consecutive samples or as an increase of concentrations to above the cut-off value. In most studies CA 125 indicated progressive disease if a prechange concentration increased by 50% (Krebs et al, 1986; Vergote et al,

1992) or by 100% (Bast et al, 1983, 1984; Fioretti et al, 1987; Panza et al, 1988; Gadducci et al, 1990, 1991; Cruickshank et al, 1992; Fioretti et al, 1992). It has also been suggested that CA 125 indicated progression if: (1) after 2 fluctuating concentrations a third concentration increased by 25% as compared to sample 1 and 2 and was confirmed by a fourth measurement, (2) there was a 50% increase over 3 samples, or (3) if there was a persistent elevation of CA 125 over 100 U/mL for more than 2 months without a 50% decrease (Rustin et al, 1993). Other investigators suggested progression criteria that were valid only during follow-up of patients where CA 125 concentrations had decreased to below the cut-off value after initial chemotherapy. According to these suggestions a positive CA 125 signal of progression was either based upon a single increment above the cut-off value (Niloff et al, 1986; van der Burg et al, 1990; Hising et al, 1991; Ward et al, 1993; Gard and Houghton, 1994), or upon an increase of concentrations to twice the upper limit of normal (Rustin et al, 1996). Thus, several empirical criteria for CA 125 progression have been applied, but none have been precise enough to eliminate false-positive results. Therefore it may be suggested, that a number of parameters should be taken into account for interpretation of CA 125 data to discriminate between variations due to the natural history of malignancy and effective therapy from those arising from other causes. Parameters such as analytical imprecision and intra-individual biological variation, which also influence marker concentrations, have not previously been considered for tumour marker assessment during monitoring of ovarian cancer. In the present study, a change in CA 125 concentrations had to exceed the variability accounted for by both biological fluctuation and analytical imprecision. The approach has been suggested by other investigators (Browning, 1987; Browning and McFarlane, 1990; Sölétormos et al, 1993b; Gion et al, 1995; Plebani et al, 1996) and tested in monitoring studies of breast cancer (Sölétormos et al, 1993*a*, 1996). Additionally, the duration of a change in concentrations and the cut-off value were also incorporated as parameters in the current proposed progression criteria. The progression criteria 1 and 2 included 2 different patterns of increasing CA 125 concentrations: a fast and a slow rise pattern (Figure 1A and 1B) reflecting the fast and indolent growth of progressive disease.

By considering the above-mentioned parameters in the progression criteria 1 and 2 the number of false-positive results was diminished but not eliminated (Table 3). CA 125 gave a false-positive prediction of progression in one patient during treatment. The patient had unusual marker profiles with considerably fluctuating CA 125 concentrations, all above the cut-off value despite clinical non-progression. It is well known that coexisting benign disease as e.g. liver cirrhosis or peritonitis, may cause elevated CA 125 concentrations (Ruibal et al, 1984; Molina et al, 1991; Collazos et al, 1992). False-positive results may also be due to an underestimation of the critical difference for the individual patient by application of the population-based average intra-individual biological variation. Assessment of the intra-individual biological variation separately for each patient would probably be more relevant, but it is difficult to perform, as it requires a collection of several consecutive measurements during steady state conditions.

Application of the progression definition based upon an increment of 2.5 times the baseline concentration confirmed by a third measurement required a greater magnitude of the critical difference as compared to the progression criteria 1 and 2 and reduced the number of false-positive results (Table 5). Generally, fewer false-positive results give higher specificity and positive predictive value, albeit at the expense of fewer true-positive results, implying lower sensitivity. Knowledge of this relationship is important, as the selection of marker assessment criteria should partly depend on whether a high sensitivity or a high specificity is considered important for the specific clinical monitoring situation. The applied criteria also have an impact on the length of lead times. The elaborated progression definition gave shorter lead times as compared to the progression criteria 1 and 2 (Table 3 and 5). However, differences in lead times of 6 days are clinically irrelevant.

Other criteria based upon a 50% or 100% increment of a prechange concentration were also taken into consideration in the present study. The definitions were, however, too unprecise producing too many false-positive CA 125 signals. The doubling of a prechange concentration of other tumour markers, CA 15.3 and CEA, was, however, successfully applied in monitoring studies of patients with breast cancer (Sölétormos et al, 1993*a*, 1996). The discrepancy concerning the utility of the doubling criterion reported by Sölétormos et al (1993*a*, 1996) as compared to the present investigation was due to the magnitude of the intra-individual biological variation of the tested markers. The intra-individual biological variation of CA 15–3 and CEA was considerably lower than the variation of CA 125 resulting in a critical difference which was lower than a doubling of concentration. Thus, the doubling of CA 15.3 and CEA concentration was always significant contrary to the doubling of CA 125 concentration.

The main purpose of this study was to evaluate the ability of CA 125 to provide reliable information about progressive disease during postoperative treatment. The results indicated that CA 125 was a reliable predictor of progressive disease. A patient with clinically suspected progression during first-line chemotherapy had a greater than 91% probability of tumour growth if CA 125 indicated progression using the progression criteria 1 and 2 (Table 3). The probability raised to 100% if the progression definition was based upon an increment of 2.5 times the baseline concentration confirmed by a third measurement (Table 5).

In conclusion CA 125 gave reliable prediction of progressive disease during monitoring of first-line chemotherapy. The present results indicate a high applicability of the progression criteria 1 and 2 as well as the criterion based upon an increment of 2.5 times the baseline concentration confirmed by a third measurement during CA 125 monitoring of patients with ovarian cancer. A further analysis of data from the follow-up period is near completion and will hopefully provide further objective information regarding the value of well defined marker progression criteria.

### ACKNOWLEDGEMENTS

We gratefully acknowledge Gordon Rustin, MD, from the Department of Medical Oncology, Mount Vernon Hospital, Middlesex, England for providing the data from the North Thames Ovary Group as the material for this study. We are also grateful to data manager Ann Nelstrop from Mount Vernon Hospital for excellent collaboration with collecting follow-up data.

### REFERENCES

Armitage P and Berry G (1994) *Statistical Methods in Medical Research*. Blackwell Scientific Publications: Oxford

 © 2001 Cancer Research Campaign

Bast RC Jr, Klug TL, St John E, Jenison E, Niloff JM, Lazarus H and Berkowitz RS (1983) A radioimmunoassay using a monoclonal antibody to monitor the course of epithelial ovarian cancer. *N Engl J Med* **309**: 883–887

Bast RC Jr, Klug TL, Schaetzl E, Lavin P, Niloff JM, Greber TF, Zurawski VR and Knapp RC (1984) Monitoring human ovarian carcinoma with a combination of CA 125, CA 19–9, and carcinoembryonic antigen. *Am J Obstet Gynecol* **149**: 553–559

Berek JS, Bertelsen K, Du Bois A, Brady MF, Carmichael J, Eisenhauer EA, Gore M, Grenman S, Hamilton TC, Hansen SW, Harper PG, Horvath G, Kaye SB, Lück HJ, Lund B, McGuire WP, Neijt JP, Ozols RF, Parmar M, Piccart-Gebhart MJ, Rosenberg P, Rustin G, Sessa C, Trope C, Tuxen MK, van Rijswijk R, Vergote IB, Vermorken JB and Willemse PHB (1999) Advanced epithelial ovarian cancer: 1998 consensus statements. *Ann Oncol* **10**: S87–S92

Boscato LM and Stuart MC (1988) Heterophilic antibodies: a problem for all immunoassays. *Clin Chem* **34**: 27–33

Browning MCK (1987) Biological variation of the breast-tumour marker CA 15–3: implications for necessary standards of performance and the interpretation of results. *Ann Clin Biochem* **24**: S1-35–S1-36

Browning MCK and McFarlane NP (1990) Objective interpretation of results for tumour markers. *J Nucl Med Allied Sci* **34**: 89–91

Cobas® Core CA 125 II EIA (1996) Roche Diagnostic Systems. Package insert

Collazos J, Genolla J and Ruibal A (1992) CA 125 serum levels in patients with non-neoplastic liver diseases. A clinical and laboratory study. *Scand J Clin Lab Invest* **52**: 201–206

Cruickshank DJ, Terry PB and Fullerton WT (1992) CA 125 -response assessment in epithelial ovarian cancer. *Int J Cancer* **51**: 58–61

Fioretti P, Gadducci A, Ferdeghini M, Bartolini T, Bianchi R and Facchini V (1987) Correlation of CA 125 and CA 19–9 serum levels with clinical course and second-look findings in patients with ovarian carcinoma. *Gynecol Oncol* **28**: 278–283

Fioretti P, Gadducci A, Ferdeghini M, Prontera C, Malagnino G, Facchini V, Marianin G and Bianchi R (1992) The concomitant determination of different serum tumor markers in epithelial ovarian cancer: relevance for monitoring the response to chemotherapy and follow-up of patients. *Gynecol Oncol* **44**: 155–160

Fraser CG and Petersen PH (1991) The importance of imprecision. *Ann Clin Biochem* **28**: 207–211

Fraser CG, Petersen PH and Larsen ML (1990) Setting analytical goals for random analytical error in specific clinical monitoring situations. *Clin Chem* **36**: 1625–1628

Gadducci A, Ferdeghini M, Ceccarini T, Prontera C, Facchini V, Bianchi R and Fioretti P (1990) A comparative evaluation of the ability of serum CA 125, CA 19–9, CA 15–3, CA 50, CA 72–4 and TATI assays in reflecting the course of disease in patients with ovarian carcinoma. *Eur J Gynaecol Oncol* **11**: 127–133

Gadducci A, Ferdeghini M, Rispoli G, Prontera C, Bianchi R and Fioretti P (1991) Comparison of tumor associated trypsin inhibitor (TATI) with CA 125 as a marker for diagnosis and monitoring of epithelial ovarian cancer. *Scand J Clin Lab Invest* **51**: 19–24

Gard GB and Houghton CRS (1994) An assessment of the value of serum CA 125 measurements in the management of epithelial ovarian carcinoma. *Gynecol Oncol* **53**: 283–289

Gion M, Barioli P, Mione R, Cappelli G, Vignati G, Fortunato A, Saracchini S, Biasioli R and Gulisano M (1995) Tumor markers in breast cancer follow-up: A potentially useful parameter still awaiting definitive assessment. *Ann Oncol* **6**: S31–S35

Hising C, Anjegard IM and Einhorn N (1991) Clinical relevance of the CA 125 assay in monitoring of ovarian cancer patients. *Am J Clin Oncol* **14**: 111–114

Krebs HB, Goplerud DR, Kilpatrick SJ, Myers MB and Hunt A (1986) Role of CA 125 as tumor marker in ovarian carcinoma. *Obstet Gynecol* **67**: 473–477

Molina R, Filella X, Bruix J, Mengual P, Bosch J, Calvet X, Jo J and Ballesta AM (1991) Cancer antigen 125 in serum and ascitic fluid of patients with liver diseases. *Clin Chem* **37**: 1379–1383

Nicholson S, Fox M, Epenetos A and Rustin G (1996) Immunoglobulin Inhibiting Reagent®: evaluation of a new method for eliminating spurious elevations in CA 125 caused by HAMA. *Int J Biol Markers* **11**: 46–49

Niloff JM, Knapp RC, Lavin PT, Malkasian GD, Berek JS, Muriel R, Whitney C, Zurawski VR and Bast RC (1986) The CA 125 assay as a predictor of clinical recurrence in epithelial ovarian cancer. *Am J Obstet Gynecol* **155**: 56–60

Panza N, Pacilio G, Campanella L, Peluso G, Battista C, Amoriello A, Utech W, Vacca C and Lombardi G (1988) Cancer antigen 125, tissue polypeptide antigen, carcinoembryonic antigen, and beta-chain human chorionic gonadotropin as serum markers of epithelial ovarian carcinoma. *Cancer* **61**: 76–83

Plebani M, Giacomini A, Beghi L, De Paoli M, Roveroni G, Galeotti F, Corsini A and Fraser CG (1996) Serum tumor markers in monitoring patients: interpretation of results using analytical and biological variation. *Anticancer Res* **16**: 2249–2252

Ruibal A, Encabo G, Martinez-Miralles E, Murcia C, Capdevila JA, Salgado A and Martinez-Vasquez JM (1984) CA 125 seric levels in non malignant pathologies. *Bull Cancer* **71**: 145–146

Rustin GJS, Nelstrop A, Stilwell J and Lambert HE (1992) Savings obtained by CA-125 measurements during therapy for ovarian carcinoma. *Eur J Cancer* **28**: 79–82

Rustin GJS, van der Burg MEL and Berek JS (1993) Tumour markers. *Ann Oncol* **4**: S71–S77

Rustin GJS, Nelstrop AE, Tuxen MK and Lambert HE (1996) Defining progression of ovarian carcinoma during follow-up according to CA 125: A North Thames Ovary Group study. *Ann Oncol* **7**: 361–364

Rustin GJ, Nelstrop AE, Crawford M, Ledermann J, Lambert HE, Coleman, Johnson J, Evans H, Brown S and Oster W (1997) Phase II trial of oral altretamine for relapsed ovarian carcinoma: evaluation of defining response by serum CA125. *J Clin Oncol* **15**: 172–176

Storm HH, Pihl J, Michelsen E and Nielsen AL (1996) *Cancer incidence in Denmark 1993*. Danish Cancer Society

Sölétormos G, Nielsen D, Schiøler V, Skovsgaard T, Winkel P, Mouridsen HT and Dombernowsky P (1993a) A novel method for monitoring high-risk breast cancer with tumor markers: CA 15.3 compared to CEA and TPA. *Ann Oncol* **4**: 861–869

Sölétormos G, Schiøler V, Nielsen D, Skovsgaard T and Dombernowsky P (1993b) Interpretation of results for tumor markers on the basis of analytical imprecision and biological variation. *Clin Chem* **39**: 2077–2083

Sölétormos G, Nielsen D, Schiøler V, Skovsgaard T and Dombernowsky P (1996) Tumor markers cancer antigen 15.3, carcinoembryonic antigen, and tissue polypeptide antigen for monitoring metastatic breast cancer during first-line chemotherapy and follow-up. *Clin Chem* **42**: 564–575

Thigpen T, Vance RB, McGuire WP, Hoskins WJ and Brady M (1995) The role of paclitaxel in the management of coelomic epithelial carcinoma of the ovary: a review with emphasis on the Gynecologic Oncology Group experience. *Semin Oncol* **22**: 23–31

Tuxen MK, Sölétormos G and Dombernowsky P (1995) Tumor markers in the management of patients with ovarian cancer. *Cancer Treat Rev* **21**: 215–245

Tuxen MK, Sölétormos G, Rustin GJS, Nelstrop AE and Dombernowsky P (2000) Biological variation and analytical imprecision of CA 125 in patients with ovarian cancer. *Scand J Clin Lab Invest* **60**: 713-722

van der Burg ME, Lammes FB and Verweij J (1990) The role of CA 125 in the early diagnosis of progressive disease in ovarian cancer. *Ann Oncol* **1**: 301–302

Vergote IB, Abeler VM, Bormer OP, Stigbrand T, Trope C and Nustad K (1992) CA 125 and placental alkaline phosphatase as serum tumor markers in epithelial ovarian carcinoma. *Tumor Biol* **13**: 168–174

Ward BG, McGuckin MA, Ramm LE, Coglan M, Sanderson B, Tripcony L and Free KE (1993) The management of ovarian carcinoma is improved by the use of cancer-associated serum antigen and CA 125 assays. *Cancer* **71**: 430–438

*© 2001 Cancer Research Campaign*
